# Do Formal Inspections Ensure that British Zoos Meet and Improve on Minimum Animal Welfare Standards?

**DOI:** 10.3390/ani3041058

**Published:** 2013-11-08

**Authors:** Chris Draper, William Browne, Stephen Harris

**Affiliations:** 1 Born Free Foundation, 3 Grove House, Foundry Lane, Horsham, West Sussex, RH13 5PL, UK; 2 School of Biological Sciences, University of Bristol, Woodland Road, Bristol, BS8 1UG, UK; E-Mail: s.harris@bristol.ac.uk; 3 School of Veterinary Sciences, University of Bristol, Lower Langford, Bristol, BS40 5DU, UK; E-Mail: william.browne@bristol.ac.uk

**Keywords:** animal welfare, captive wild animals, enforcement, government inspections, improving standards, legislative oversight, local authority, Zoo Licensing Act

## Abstract

**Simple Summary:**

Key aims of the formal inspections of British zoos are to assess compliance with minimum standards of animal welfare and promote improvements in animal care and husbandry. We compared reports from two consecutive inspections of 136 British zoos to see whether these goals were being achieved. Most zoos did not meet all the minimum animal welfare standards and there was no clear evidence of improving levels of compliance with standards associated with the Zoo Licensing Act 1981. The current system of licensing and inspection does not ensure that British zoos meet and maintain, let alone exceed, the minimum animal welfare standards.

**Abstract:**

We analysed two consecutive inspection reports for each of 136 British zoos made by government-appointed inspectors between 2005 and 2011 to assess how well British zoos were complying with minimum animal welfare standards; median interval between inspections was 1,107 days. There was no conclusive evidence for overall improvements in the levels of compliance by British zoos. Having the same zoo inspector at both inspections affected the outcome of an inspection; animal welfare criteria were more likely to be assessed as unchanged if the same inspector was present on both inspections. This, and erratic decisions as to whether a criterion applied to a particular zoo, suggest inconsistency in assessments between inspectors. Zoos that were members of a professional association (BIAZA) did not differ significantly from non-members in the overall number of criteria assessed as substandard at the second inspection but were more likely to meet the standards on both inspections and less likely to have criteria remaining substandard. Lack of consistency between inspectors, and the high proportion of zoos failing to meet minimum animal welfare standards nearly thirty years after the Zoo Licensing Act came into force, suggest that the current system of licensing and inspection is not meeting key objectives and requires revision.

## 1. Introduction

While there is intense public interest in the welfare of captive animals, the zoo community has been slow to establish consistent standards for assessing animal welfare [1]. The USA-based Association of Zoos and Aquariums (AZA) was established in 1924 but only formed its Animal Welfare Committee in 2000. In the UK, the Zoo Licensing Act 1981 (ZLA) was one of the first pieces of legislation in Europe that established a system of zoo inspections [2]. It came into effect in 1984, and the subsequent European Zoos Directive (Council Directive 1999/22/EC), which requires that European Union member states implement measures for the licensing and inspection of zoos, was modelled to a large extent on the ZLA [3]. Inspections of British zoos generally last for one day [4], and are designed to monitor overall standards rather than assess the welfare of individual animals. However, designing animal welfare assessments that are quick and easy to undertake, but still provide useful data, poses a significant challenge [1]. While the British system of zoo inspections has been in place for nearly three decades, the data from these inspections have not been compiled or analysed centrally, and so it is unclear whether the inspection process is monitoring animal welfare standards and/or leading to improvements. Periodic reviews of the inspection process are key to improving animal welfare standards, both in British zoos and the zoo community more generally. 

There are approximately 273 licensed zoos in Britain (230 in England, 28 in Scotland, 15 in Wales) [5,6,7,8]. The ZLA requires that all British zoos are inspected and licensed; formal inspections are undertaken by Local Authorities in consultation with government-appointed Zoo Inspectors (ZIs) to grant or renew a licence (renewal inspections) and at least once during the licence period (periodic inspections); see [4] for details. A key function of ZIs is to “*provide consistent advice to local authorities, with the aim of monitoring and promoting .... high standards of animal care and husbandry in zoos*” [9]. The ZLA is supplemented by the Secretary of State’s Standards of Modern Zoo Practice (SSSMZP); these provide guidance to zoo operators and inspectors on the Act’s implementation and requirements, and represent the “*minimum Standards that zoos … are expected to meet*” [10]. The SSSMZP also “*function as an interpretation of the law and provide criteria against which zoos’ compliance with the law will be judged*” [11]. ZIs may also use the inspection to encourage the zoo to aspire to standards higher than these minimum requirements [12], although responsibility for implementing improvements lies with the zoos themselves, not the ZIs. However, formal inspections represent an opportunity for competent (as decided by the Secretary of State) independent experts to assess zoos and make recommendations on the basis of up-to-date knowledge of animal welfare, legislation and the prevailing standards expected of zoos [4,13].

Formal inspections also provide opportunities for ZIs to recommend that improvements be made: Section 10(5) of the ZLA states that, following an inspection, ZIs “*shall send their report to the local authority, and the report may include advice on the keeping of records and recommendations for any practicable improvements designed to bring any features of the zoo up to the normal standards of modern zoo practice*”. A Local Authority can attach conditions to a zoo’s licence when they consider this necessary or desirable for ensuring the proper conduct of the zoo during the period of the licence (Section 5(3), ZLA). Any zoo not meeting the required minimum standards should improve in order to do so, or face refusal to renew their licence or closure proceedings. However, Local Authorities should not normally refuse a licence “*where there were reasonable prospects that improvements would take place*” [14]. There are no records on how frequently zoos have had their licence renewal refused or faced closure proceedings; while at least 60 UK facilities have closed since 2000 [15], most of these are believed to have closed for financial or operational reasons. 

The British and Irish Association of Zoos and Aquariums (BIAZA) is a professional membership body formed in 1966 as the Federation of Zoological Gardens of Great Britain and Ireland. BIAZA claims that it “*leads and supports its members to achieve the highest standards of animal care and welfare*” and that its members “*must do more than comply with zoo legislation*” [16]. It has 102 full and provisional members across Britain and Ireland [17]. Thus both the inspection process and membership of BIAZA should lead to animal care and welfare practices that exceed the minimum standards defined for British zoos. 

In this paper we build on the results of an earlier analysis of zoo inspection reports from 2005 to 2008 [4]. As zoos are inspected by ZIs approximately every 3–4 years, subsequent inspection reports have become available for many of the zoos included in [4], enabling us to examine whether zoos that failed to meet the minimum animal welfare standards at the first inspection had improved by the subsequent inspection, and conversely whether zoos that met the minimum standards at the first inspection did not meet them at the second inspection. If the licensing and inspection process is effective, we anticipated seeing more zoos assessed as meeting the standards at the second inspection. In particular, we wanted to determine if substandard assessments are repeated from one inspection to the next or whether problems identified by ZIs are addressed by zoos. We also examine whether the type of zoo and membership of BIAZA influence changes in assessments by ZIs, and whether having the same ZI for consecutive inspections influences the outcome of an inspection.

## 2. Experimental Section

For details of the animal welfare assessments, and data collection for the first inspections, see [4]. We requested reports from the subsequent inspections of the same zoos from Local Authorities between April and December 2012 and information from 47 questions in Section 1 to 5 (Appendix 1), corresponding approximately to the Five Freedoms, were collated as described previously [4]. One question was not included in the analysis (1.6 “*Is feeding by visitors permitted?*”) as it was less directly relevant to animal welfare than other questions. A supplementary question (1.6.a) required ZIs to assess whether feeding by the public is properly controlled; this is directly relevant to animal welfare and was included in the analyses.

For both inspections, a zoo was considered substandard for a criterion when the ZIs marked NO, or marked YES but then recommended that a condition for improvement relating to that criterion should be attached to the licence. We did not include comments, remarks and recommendations for improvements as substandard when these were not associated with recommendations that conditions be attached to the licence [4]. Where we state that a zoo(s) met all the standards, this only includes the relevant standards. So, for instance, some answers may have been N/A when one or more criteria did not apply to a zoo (see Section 3).

Zoos were categorised by type following [5]. Membership of BIAZA was determined as described in [4] and subsequent BIAZA annual reports [17,18,19]. The names of the inspectors were obtained from the zoo inspection report forms; for 26 zoos these were not available because they were redacted on the first and/or second inspection report by the Local Authority.

### Statistical Analyses

The data were inputted into Microsoft Excel^©^. Multivariate analysis was considered unsuitable due to the limitations of the data (see Section 4.1) so we only tried to identify general patterns. Since the data were not normally distributed, we used non-parametric statistics to identify factors associated with animal welfare assessment in zoos. Data were analysed using SPSS^®^ v. 19.0. Results were considered statistically significant when *P* < 0.05.

## 3. Results and Discussion

We obtained subsequent (second) inspection report forms for 136 of the 192 zoos (71%) included in [4]. These constituted approximately 50% of the licensed zoos in Britain. First (original) inspections were made between May 2005 and December 2008, second inspections between January 2009 and December 2012. These included at least 39 zoos with full licences (*i.e.*, without dispensations on grounds of size of collection or species kept), BIAZA members and non-members, and a range of types of zoo. Outcomes of the two inspections are summarised in Appendix 1. 

**Table 1:** Comparison of zoo assessments at first and second inspections.

		First inspection		Total
		Met all standards	Did not meet all standards	
**Second inspection**	**Met all standards**	22	22	44
	**Did not meet all standards**	13	79	92
**Total**		35	101	136

The interval between first and second inspections for 133 zoos ranged from 525 to 2,254 days (median = 1,107 days); the interval between inspections could not be determined for three zoos as the dates were missing or incomplete. The interval between first and second inspections was not associated with the number of criteria rated substandard at the second inspection (Spearman rank correlation, n = 133, r = 0.109, *P* > 0.05). There was no evidence of second inspections lasting more than one day, and on three occasions the same ZI inspected two zoos on the same day *i.e.*, 6/108 zoos (6%) for which we had the names of the ZIs and dates for both inspections. Of the 136 zoos, 35 (26%) met all the standards at the first inspection, 44 (32%) at the second inspection. Of the 79 zoos (58%) that did not meet all the standards at both inspections, 48 (35% of all zoos) were judged substandard on at least one of the same criteria at both visits (Table 1).

Of the 44 zoos assessed as meeting all the standards at the second inspection, 22 (50%) had met all the standards at the first inspection, whereas 13 of the 35 zoos (37%) that met all the standards in the first inspection were considered substandard on one or more criteria in the second inspection. Across all zoos, 502/6,392 (7.9%) individual criteria were assessed as substandard at the first inspection, 394/6,392 (6.2%) at the second inspection. Of the substandard assessments, 357 were marked as “No” and 145 marked as “Yes” but with recommended conditions at the first inspection compared with 256 and 138 respectively at the second inspection. At the first inspection, 5,373 (84.1%) criteria were assessed as meeting the standards, 5,568 (87.1%) at the second inspection, and 517 (8.1%) criteria were marked as N/A or left blank at the first inspection, 430 (6.7%) at the second inspection.

While there was a small increase in the proportion of zoos meeting all the standards between the two inspections, the majority of zoos were assessed as not meeting all the standards at the second inspection (Table 2) and more than a third of the zoos that met all the standards at the first inspection no longer met them all at the second inspection. Furthermore, 35% of zoos had at least one criterion that was assessed as substandard at both inspections. In these cases, there was not usually enough information in the reports to determine whether the same specific issue was detected at both inspections (e.g., a problem with a particular animal or enclosure), or whether it was a more general issue.

**Table 2:** The number of criteria per zoo for each of nine possible outcomes at the first and second inspections: n = 136 zoos.

		First Inspection		
		Standards met	Substandard	Blank or N/A
**Second Inspection**	**Standards met**	Median (IQR): 39 (8) Minimum: 16 Maximum: 46	Median (IQR): 2 (4) Minimum: 0 Maximum: 18	Median (IQR): 1 (2) Minimum: 0 Maximum: 10
	**Substandard**	Median (IQR): 1 (2.75) Minimum: 0 Maximum: 12	Median (IQR): 0 (1) Minimum: 0 Maximum: 9	Median (IQR): 0 (0) Minimum: 0 Maximum: 2
	**Blank or N/A**	Median (IQR): 0 (1) Minimum: 0 Maximum: 3	Median (IQR): 0 (0) Minimum: 0 Maximum: 1	Median (IQR): 3 (2) Minimum: 0 Maximum: 9

There are several criteria where “N/A” might be expected as a response to a question for some zoos. For example, question 2.5 “*Are backup facilities for life support systems adequate?*” could only apply to specialist facilities such as Aquariums, and question 3.7 “*Is darting equipment satisfactory?*” may not apply to zoos keeping species for which darting is not used. However, for other questions N/A was an uninformative response. For example, of the 59 zoos that were assessed as either meeting the standards or being substandard at first inspection on criteria such as 1.6.a (*If* [feeding by visitors is permitted], *is it properly controlled?*), 14 (23.7%) were subsequently marked “N/A” or left blank at the second inspection. While this could reflect changes in practice at the zoos between the inspections, rendering the question no longer relevant, the number of zoos involved is surprising (Table 2). In other cases it was impossible to understand why some questions were considered relevant at one inspection and not at the other. For 10 zoos, for example, the assessment for question 5.2 (*Is physical contact between animals and the public consistent with the animals’ welfare?*) changed from blank or N/A at first inspection to being assessed as either meeting the standard or being substandard at the second inspection, while 8 zoos changed from either meeting the standard or being substandard at the first inspection to being left blank or assessed as N/A at the second inspection.

In 81 zoos (60%), one or more criteria that had been marked as “N/A” or left blank at first inspection were recorded as meeting the standards at the second inspection, while in 22 zoos (16%) one or more criteria that had been marked as “N/A” or left blank at first inspection were assessed as substandard at the second inspection. Across all 136 zoos, of the 517 individual criteria that had been marked N/A or left blank at the first inspection, 163 (32%) were judged to meet the standards at the second inspection and 30 (6%) were substandard. Comparing the number of criteria assessed as substandard on each of the two inspections, the average number per zoo considered substandard on the first inspection was 3.76/47 (8%), with one zoo having 19 of the 47 criteria (40%) considered substandard; comparable figures for the second inspection were 2.82 (6%) and 13 (28%). There was a statistically significant reduction in the number of criteria considered substandard (Wilcoxon signed ranks test, Z = −2.33, *P* < 0.05), suggesting an overall improvement in welfare standards, but there was no significant change when comparing whether individual zoos have less or more substandard criteria (Sign test, Z = −1.50, *P* > 0.05) (Table 3).

**Table 3:** Number of criteria assessed substandard per zoo for each section (see Appendix 1) on the first and second inspections; n = 136 zoos.

		First inspection			Second inspection		
Section	No. of criteria assessed per zoo	Median no. substandard per zoo	Maximum	Minimum	Median no. substandard per zoo	Maximum	Minimum
**1. Provision of food and water**	8	0	6	0	0	3	0
**2. Provision of a suitable environment**	13	0	6	0	0	5	0
**3. Provision of animal health care**	22	1	12	0	1	8	0
**4. Provision of an opportunity to express most natural behaviour**	1	0	1	0	0	1	0
**5. Provision of protection from fear and distress**	3	0	2	0	0	2	0
**Overall**	47	2	19	0	2	13	0

There was a statistically significant effect of the type of zoo on the total number of criteria continuing to be substandard between the first and second inspections (Table 4). Zoos classified as “Other Bird” and “Farm Park” performed least well, whereas zoos classified as “Aquarium” performed best. This reinforces an earlier analysis [4] which raised concerns about the animal welfare standards in “Farm Park” and “Other Bird” zoos. 

**Table 4:** Effect of type of zoo on total number of criteria continuing to be substandard between first and second inspections. The type of zoo is based on [5]; higher mean ranks indicate that more criteria were assessed as being substandard at both the first and second inspection. Since there was only one “Big Cat” zoo and one “Reptile Amphibian” zoo in the sample, they were excluded from the analyses. Assessments of different types of zoo were compared with Kruskal-Wallis tests: n = 134 zoos.

Type of zoo	n	Mean rank
Farm Park	15	81.87
Other Bird	11	76.05
Bird of Prey	20	75.65
General Mixed	55	68.86
Other	8	53.19
Invertebrate	6	52.58
Aquarium	19	49.42
Significance		X^2^ = 13.302, *P* < 0.05

Membership of BIAZA is not constant: using both (a) the BIAZA membership list employed in [4], and (b) a list reflecting subsequent membership changes [17,18,19], there was no significant difference in the number of criteria assessed as substandard at second inspection between zoos that were members of BIAZA ((a) n = 60; (b) n = 66) and those that were not ((a) n = 76; (b) n = 70) (Mann-Whitney, (a) U = 2,068.5, Z = −0.946, *P* > 0.05; (b) U = 2,158.5, Z = −0.673, *P* > 0.05). BIAZA members did not differ significantly from non-members in the overall number of criteria assessed as substandard at the second inspection. However, there were significant differences between BIAZA members and non-members in the number of criteria remaining compliant between first and second inspections (Mann-Whitney, (a) U = 1,411, Z = −3.817, *P* < 0.005; (b) U = 1,446, Z = −3.770, *P* < 0.005) and number of criteria remaining substandard between first and second inspections (Mann-Whitney, (a) U = 1,845, Z = −2.237, *P* < 0.05; (b) U = 1,769, Z = −2.763, *P* < 0.05). Thus zoos that were members of BIAZA were more likely to meet the standards on both inspections and less likely to have criteria remaining substandard.

At least 53 different combinations of ZIs undertook the second inspections. Of the 110 zoos where the names of the ZIs were available for both inspections, at least one of the same ZIs undertook both inspections in 46 zoos (42%). The presence of at least one of the same ZIs at both inspections was significantly associated with the number of criteria remaining the same at both inspections *i.e.*, either continuing to meet the standards or continuing to be substandard (Mann-Whitney, U = 1,061, Z = −2.497, *P* < 0.05). 

## 4. Conclusions

We build on an earlier analysis of the zoo inspection process in Britain [4] to see whether formal zoo inspections are leading to higher standards of animal welfare in zoos, a key aspiration of the inspection process [9]. The outcome of a zoo inspection could reflect changes in conditions within the zoo, either in response to or independent of the inspections, and/or variability between inspections and/or ZIs. It could, for instance, be argued that animal welfare standards and the benchmarks against which zoos are assessed inevitably change and improve over time, leading to zoos that do not change gradually falling below the standards. However, the SSSMZP have not changed significantly with respect to animal welfare provisions since 2004; a technical update in 2012 added standards for elephant keeping but only one zoo in the sample, which does not keep elephants, was inspected after that date, so the SSSMZP standards were consistent between the two inspections. It is also possible that there have been background changes in practices related to, but not specified in, the standards. For example, advances in veterinary technology might mean that onsite veterinary facilities require regular improvements in order to be considered adequate (question 3.8). However, since many of the criteria assessed relate to basic provisions for animal husbandry and housing, which do not change rapidly, it is unlikely that underlying advances in best practice are the cause of many zoos being assessed as substandard. Moreover, if there are advances in the minimum standards of animal care and husbandry, it is a zoo’s responsibility to implement them. Each zoo is also responsible for addressing other factors that may influence its ability to deliver adequate animal welfare, such as enclosures and materials aging, the needs of animals changing during their lifetimes, changes in the number and types of animals in the collection, and the financial consequences of changing visitor numbers. 

The zoos included in this study ranged in size and type, and comparisons between them can be fraught with difficulties. However, all zoos must comply with the ZLA and the associated standards. If the SSSMZP are the minima that zoos are expected to meet [10,11], it might reasonably be expected that an overwhelming majority of zoos should meet all the standards, especially since the SSSMZP “*provide criteria against which zoos’ compliance with the law will be judged*” [11]. Similarly, if the zoo licensing and inspection process is functional, substandard zoos should improve over time and the standards in other zoos should be maintained [20]. If a zoo does not comply with one or more conditions attached to their license, local authorities are required to issue a direction requiring compliance with the condition(s), unless the zoo closes (Section 16A, ZLA) [10]. This direction stipulates the steps required to comply with the standards and the period for compliance.

It is surprising, therefore, to see so many zoos being assessed as not meeting the standard for a particular criterion and/or having additional conditions for that criterion attached to the licence at both inspections. In the period 2005–2008, ZIs reported that 24% of zoos were not complying with one or more of the pre-existing conditions on their licence, *i.e.*, the necessary improvements to meet the minimum standards were not being implemented by the zoos [4]. While “*Zoos pursue a variety of aims, including conservation, education, research and the provision of recreation, and they have to operate within financial and other resource constraints*” [13], the welfare of the animals on display should be a resource priority, and there is no provision in the ZLA for exemptions from meeting the standards on financial or other grounds.

### 4.1. Consistency of the Data

Consistency of assessments is integral to ensuring that zoos meet the minimum standards of animal welfare, and that animal welfare standards in zoos improve. A number of issues identified by this analysis raise cause for concern. Despite a minimum of 53 different combinations of ZIs undertaking the second round of inspections, at least one of the same ZIs undertook both inspections in 42% of those zoos where the names of inspectors were available for both inspections. While there may be logistical reasons for this (e.g., it may be practical to use a local ZI), the dual approach of employing some different and some “repeat” inspectors is of concern since assessments of individual criteria were more likely to remain the same if at least one ZI attended both inspections. Since having different ZIs on each inspection was associated with a higher probability that welfare criteria would be assessed differently, this suggests inconsistency of assessments between inspectors, which may at least in part be due to the vague nature of the questions and the lack of clarity as to when to mark a criterion as substandard [4]. The lack of consistency between ZIs is also reflected in the use of “N/A” or leaving individual criteria blank, since these answers are not consistent between inspections.

The lack of consistency in the way the criteria appear to be assessed, including whether a criterion even applies to a particular zoo, undermines the value of the data collected. This and the earlier analysis [4] highlight the need for more rigorous assessments, made in an electronic format to facilitate future analyses. Until better quality data are available, it is hard to monitor changes in zoo animal welfare standards, and identify areas where improvements are needed. Central analyses of general trends across zoos should be complemented by more detailed studies to assess the influence that animal care recommendations have on the welfare of individual animals [12].

### 4.2. Animal Welfare Implications

There is a myriad of legislation worldwide relating to zoos but little uniformity in the provisions, procedures, implementing authorities or enforcement [20]. The ZLA was a landmark piece of legislation and the UK is still one of the few countries with specific legislation laying out standards for zoos [20]. The inspection process, the maintenance of standards and the implementation of improvements are key to ensuring high standards of animal welfare in zoos. Currently, British zoos are only legally required to meet minimum standards, although the aspiration of both the inspection process and BIAZA is to improve standards above these minima. While it is important to ensure that minimum legal requirements are being met and exceeded if a zoo is to provide good animal welfare [21], 6.2% of the minimum animal welfare standards were not being met in British zoos 30 years after the ZLA was enacted. Our data also suggest that animal welfare in British zoos, or at least the inputs associated with animal welfare, may not necessarily improve following a formal inspection and may even decline. The current system of legislation and oversight does not ensure that zoos in Britain meet and maintain the minimum standards of animal welfare required under the ZLA, nor lead to improvements in standards of animal care and husbandry over time. Since the continuing existence of zoos in Britain and elsewhere can only be justified ethically if they guarantee the welfare of their animals [22], lessons learnt from the implementation of the ZLA in the UK should guide the development other national schemes to monitor the welfare of zoo animals. 

## Appendix 1

**Table:** Criteria relating to animal welfare that were assessed by ZIs. The figures show the number of zoos assessed in each category at the first and second inspections: n = 136 zoos.

1st inspection 2nd inspection	Standard met Standard met	Standard met Sub- standard	Sub- standard Standard met	Sub- standard Sub- standard	Blank or N/A Standard met	Blank or N/A Sub- standard	Blank or N/A Blank or N/A	Standard met or sub-standard Blank or N/A
***Section 1. Provision of food and water***								
**1.1 Is each animal provided with a high standard of nutrition?**	129	3	4	0	0	0	0	0
**1.2 Is food and drink appropriate for the species/individual supplied?**	128	3	5	0	0	0	0	0
**1.3 Are supplies of food and water:** **(a) kept hygienically?** **(b) prepared hygienically?** **(c) supplied to the animal hygienically?**	115 102 126	9 10 2	10 19 5	2 4 0	0 0 1	0 1 0	0 0 0	0 0 2
**1.4 Has natural feeding behaviour been adequately considered to ensure that all animals have access to food and drink?**	128	1	5	0	1	0	0	1
**1.5 Are feeding methods safe for staff and animals?**	131	3	2	0	0	0	0	0
**[1.6 Is feeding by visitors permitted?]** **(a) If YES, is it properly controlled?**	[45] 38	[11] 2	[17] 5	[57] 0	[2] 20	[3] 0	[0] 57	[1] 14
***Section 2. Provision of a suitable environment***								
**2.1 Is each animal provided with an environment well adapted to meet the physical, psychological and social needs of the species to which it belongs?**	104	12	11	7	1	0	0	1
**2.2 Are the following environmental parameters appropriate:** **(a) temperature?** **(b) ventilation?** **(c) lighting?** **(d) noise levels?** **(e) any other environmental parameters?**	120 123 123 130 105	6 1 4 0 5	7 4 5 2 7	2 1 1 0 1	1 7 2 4 11	0 0 1 0 1	0 0 0 0 2	0 0 0 0 4
**2.3 Do animal enclosures have sufficient shelter?**	121	6	6	2	1	0	0	0
**2.4 Do animal enclosures provide sufficient space?**	123	3	7	2	1	0	0	0
**2.5 Are backup facilities for life support systems adequate?**	85	3	7	0	16	1	11	13
**2.6 Is the cleaning of the accommodation satisfactory?**	124	5	5	1	0	0	0	1
**2.7 Is the standard of maintenance adequate for:** **(a) the buildings?** **(b) the fences?**	103 100	12 7	11 9	7 8	0 3	0 1	0 3	3 5
**2.8 Is all drainage effective and safe?**	126	1	8	0	0	0	0	1
***Section 3. Provision of animal health care***								
**3.1 Is each animal provided with a high standard of animal husbandry?**	127	4	4	0	1	0	0	0
**3.2 Do all animals on display to the public appear to be in good health?**	129	4	3	0	0	0	0	0
**3.3 Are observations of condition and health made and recorded?**	121	3	9	1	2	0	0	0
**3.4 Do all animals receive prompt and appropriate attention when problems are noted?**	130	1	4	0	1	0	0	0
**3.5 Are enclosures designed and operated in such a way that social interaction problems are avoided?**	126	1	4	1	1	0	1	2
**3.6 Are catch-up and restraint facilities adequate?**	125	3	2	0	0	1	1	4
**3.7 Is darting equipment satisfactory?**	43	1	2	1	13	5	61	10
**3.8 Are on-site veterinary facilities adequate?**	94	9	15	2	6	2	7	1
**3.9 Is each animal provided with a developed programme of preventative and curative veterinary care and nutrition?**	97	12	11	14	1	0	1	0
**3.10 Is a satisfactory programme of preventative and curative veterinary care established and maintained?**	91	15	20	9	0	0	0	1
**3.11 Is there a system for the regular review of clinical and pathological records?**	94	5	17	15	1	1	0	3
**3.12 Are appropriate veterinary records kept?**	98	9	13	12	2	1	0	1
**3.13 Are medicines correctly kept?**	96	14	7	3	4	0	8	4
**3.14 Are controlled drugs used and recorded satisfactorily?**	33	1	2	0	8	6	75	11
**3.15 Are appropriate antidotes available?**	20	0	1	0	10	3	94	8
**3.16 Are post mortem examination arrangements satisfactory?**	92	11	15	15	3	0	0	0
**3.17 Is adequate reserve accommodation** **available for isolation of animals for:** **(a) assessment?** **(b) treatment?** **(c) recovery?** **(d) quarantine (where required)?**	113 111 113 93	5 4 3 3	13 13 12 17	1 3 3 3	4 5 5 10	0 0 0 4	0 0 0 1	0 0 0 5
**3.18 Are satisfactory measures in place to prevent the intrusion of pests and vermin into the zoo premises?**	117	8	6	4	1	0	0	0
**3.19 Does it appear that general sanitation and pest control are effective?**	121	4	5	1	1	1	0	3
***Section 4. Provision of an opportunity to express most normal behaviour***								
**4.1 Does accommodation appear adequately to meet the biological and behavioural needs of the animals?**	115	9	8	3	1	0	0	0
***Section 5. Provision of protection from fear and distress***								
**5.1 Are the animals handled only by or under the supervision of appropriately experienced staff?**	128	1	4	1	1	0	0	1
**5.2 Is physical contact between animals and the public consistent with the animals’ welfare?**	109	3	3	1	9	1	2	8
**5.3 Are interactions between the animals such that they are not excessively stressful?**	126	3	2	1	4	0	0	0
